# The Subpolar North Atlantic Ocean Heat Content Variability and its Decomposition

**DOI:** 10.1038/s41598-017-14158-6

**Published:** 2017-10-23

**Authors:** Weiwei Zhang, Xiao-Hai Yan

**Affiliations:** 1 State Key Laboratory of Marine Environmental Science, Xiamen University, Xiamen, 361005 China; 2 College of Earth, Ocean, and Environment, Newark, United States; 3 College of Ocean and Earth Sciences, Xiamen University, Xiamen, 361005 China; 4 University of Delaware, Xiamen University Joint Institute for Coastal Research and Management, Newark, United States

## Abstract

The Subpolar North Atlantic (SPNA) is one of the most important areas to global climate because its ocean heat content (OHC) is highly correlated with the Atlantic Meridional Overturning Circulation (AMOC), and its circulation strength affects the salt transport by the AMOC, which in turn feeds and sustains the strength of the AMOC. Moreover, the recent global surface warming “hiatus” may be attributed to the SPNA as one of the major planetary heat sinks. Although almost synchronized before 1996, the OHC has greater spatial disparities afterwards, which cannot be explained as driven by the North Atlantic Oscillation (NAO). Temperature decomposition reveals that the western SPNA OHC is mainly determined by the along isopycnal changes, while in the eastern SPNA along isopycnal changes and isopycnal undulation are both important. Further analysis indicates that heat flux dominates the western SPNA OHC, but in the eastern SPNA wind forcing affects the OHC significantly. It is worth noting that the along isopycnal OHC changes can also induce heaving, thus the observed heaving domination in global oceans cannot mask the extra heat in the ocean during the recent “hiatus”.

## Introduction

The global mean surface temperature increase is observed to slowdown from 1998 to 2013 despite increasing greenhouse gas concentration^1–3^. Different mechanisms are proposed to explain the occurrence of the global warming “hiatus”, they are either external radiative forcing changes^4–6^, internal variability and subsurface ocean warming^2,7–9^. Nonetheless, the “hiatus” is merely a redistribution of heat in the earth’s system^1^, although it is not conclusive where most of the heat is stored away from the surface. Surface and subsurface temperature patterns have been identified during “hiatus”. For instance, the tropical Pacific surface cooled the most^7^, the Indian Ocean stored most of the heat at 100–300 m^10^. While the Atlantic and Southern Oceans at 300–1500 m depth warmed the most during the hiatus^3^. Model studies showed significant weakening of the Atlantic Meridional Overturning Circulation (AMOC) and the North Atlantic convection during “hiatus”^2^.

The Subpolar North Atlantic (SPNA) Ocean Heat Content (OHC) is highly correlated with the AMOC^11^, whose weakening is also crucial to the “hiatus”^2,3^. Before 2000, the SPNA was cooling in general comparing to the period before 1970s^12^. However, the warming of the upper 1000 m occurs since mid-1990s in the Subpolar Gyre (SPG)^13^, except strong convective cooling in the western SPNA in 2008^14^. Due to weakened winter deep convections, the western SPNA winter cooling is weakened^2^. Meanwhile, the western SPNA gains heat when the lateral eddy heat flux from the warm boundary current enters the interior^14,15^. Both the weakened convective cooling and the continuous heat flux from the boundary cause the gradual warming of the western SPG since mid-1990s. On the other hand, the sudden warming of the SPG in 1995 may be caused by SPG shrinking due to sudden switch of NAO from positive to negative^16^, or due to the East Atlantic Pattern^17,18^. Or the sudden warming is a delayed response to the AMOC, which has been strengthening during the period of NAO positive phase, and once the SPG shrinks, the enhanced northward heat flux can enter the SPG^19^.

The temperature change can be decomposed into two components^20^. One is the along isopycnal temperature change, and is termed “spice” or water mass change. The other is the temperature change due to the undulation of the isopycnal, and is termed “heaving”. Heaving and spice components are found to play different roles for different time period. For instance, some studies found heaving to be dominating the temperature variability in the North Atlantic or the SPNA^21,22^, while others found dominating spice component during the cooling of 1980–2000 compared to 1950–1970 in the SPNA^12^. The differences originate from several sources. First, either reanalysis data^21^ or model output^12^ were used. Second, the subtropical North Atlantic is heaving dominated, and due to its larger area than the SPG, so the heaving component is dominating^21^. Third, when investigating the multidecadal change between 1995–2009 and 1961–1975, different datasets have shown little consistency in the SPG region on which component is more important^21^.

Here we focus on the OHC variability in the SPNA since 1956, based on the World Ocean Atlas (WOA) dataset, objectively analyzed ocean temperature and salinity observations. A temperature decomposition method is used to reveal possible causes to the OHC changes, such as the remote or local heat flux, the gyre circulation strengthening or weakening, caused by the variability of the wind stress forcing. Although the observations are scarce before the Argo era, by using five years of data to map one single year, the WOA data is appropriate for the decomposition (Figure S1).

## Ocean Heat Content Variability

**Figure 1:**
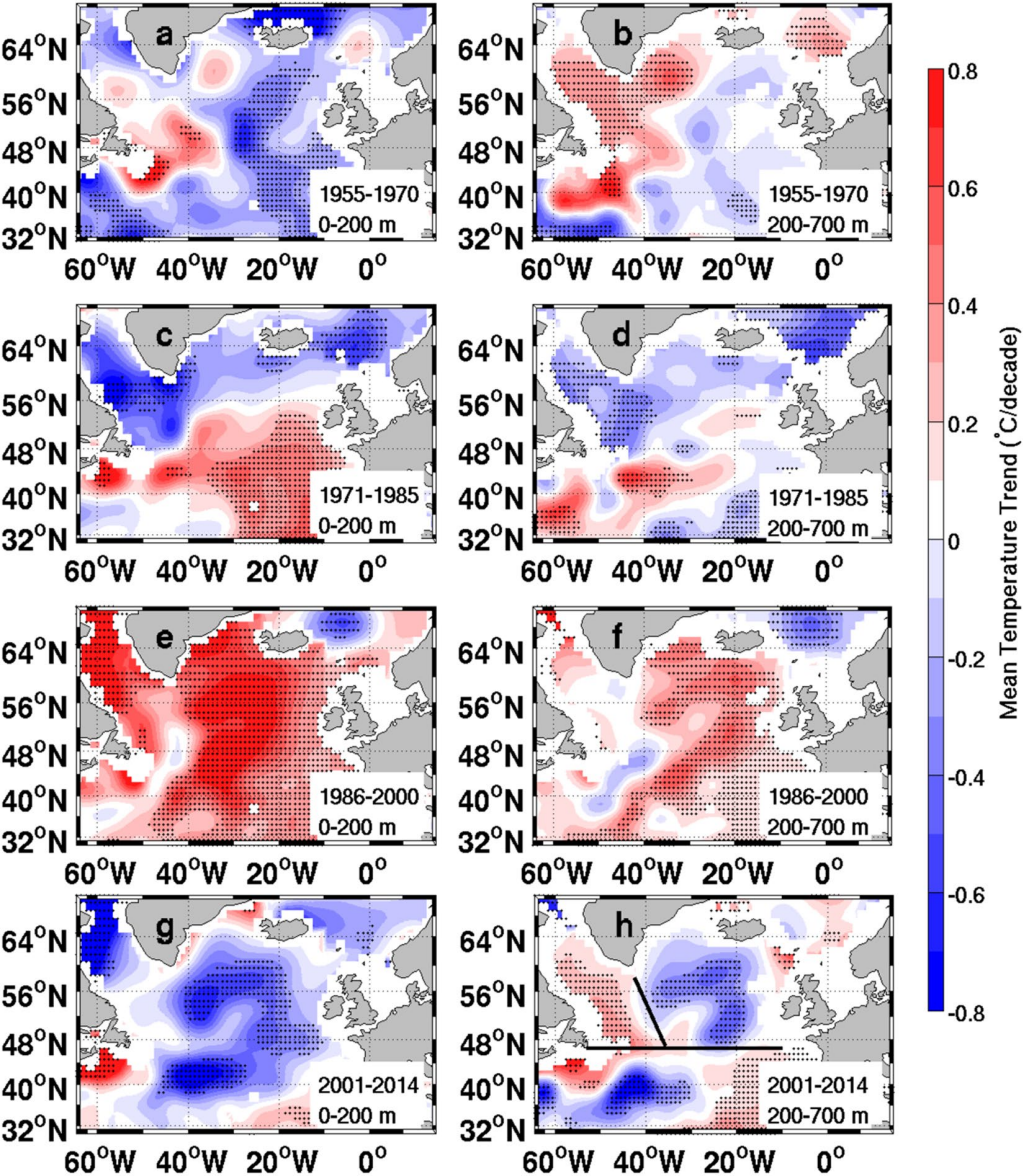
The vertically averaged temperature trend (unit: °C/decade) in the Subpolar North Atlantic. The linear trends of the temperature for different time periods are calculated for two depth ranges: 0–200 m and 200–700 m. The stipplings indicate that the fitted linear trend is above 95% confidence level. The SPNA is divided into the eastern and western sections due to the apparent opposite trends in the most recent decade or so (**h**). The map in this figure is created using the m_coast function from the m_map toolbox v1.4 for Matlab (https://www.eoas.ubc.ca/~rich/map.html).

**Figure 2:**
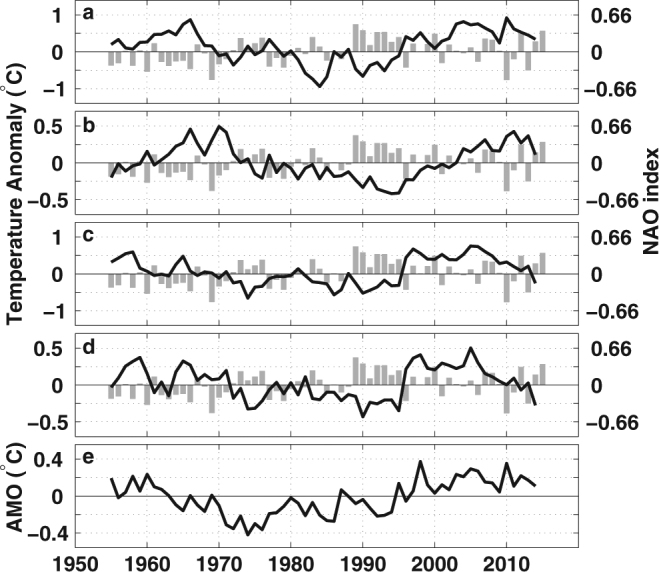
The time series of the area mean temperature anomaly, the North Atlantic Oscillation (NAO) index and the Atlantic Multidecadal Oscillation index. The western SPNA temperature anomaly at 0–200 m (**a**) and 200–700 m (**b**) are overlapped with the NAO index, and the same for (**c)** and (**d**), but for the eastern SPNA. The AMO index time series is shown in (**e**).

Figure 1 shows the vertically averaged temperature trend in two layers: 0–200 m and 200–700 m. Overall the SPNA has strong decadal variability (Fig. 1a–d), and the surface 200 m layer has similar temperature trend as the 200–700 m layer. Numerical experiments have tested the effect of atmospheric forcings on the OHC changes in the North Atlantic, and found buoyancy forcing variability is crucial in the SPNA^12^. The positive NAO is usually associated with cooler SPNA and warmer Subtropical gyre^12^, which is also confirmed in this study (Fig. 2a–d), and the correlation coefficient between the subpolar gyre water and the NAO index is −0.43 (p = 0.00062). In the 200–700 m layer, large portion of the SPNA area has the same sign of temperature trends (Fig. 1b,d,f). Roughly the “hiatus” period, the western and eastern SPNA show opposite temperature trends (Fig. 1h), indicating different physical mechanisms for OHC changes in the two regions. Previous study has shown that the western and the eastern SPNA have different responses to the positive NAO conditions from 1988 to 1995^23^. Thus, we will analyze the OHC changes separately in the western and eastern SPNA. The areas are chosen as where the temperature trends in the 200–700 m are above 95% confidence level (Fig. 1h).

The time series of the eastern SPNA and the western SPNA temperatures show similar decadal variability: warmer during the 1960s and the “hiatus” period, and cooler from 1970s to mid 1990s (Fig. 2a–d). The warm period of the subsurface western SPNA in the 1960s and early 1970s (Fig. 2b), which is likely due to the “Great Salinity Anomaly”^24^, also coincides with an earlier “hiatus”^3,25^. The eastern SPNA shows a sudden warming in 1995, and stays warm until 2005. It becomes cooler for about 1 °C afterwards in both layers (Fig. 2c,d). The surface and subsurface cooling rate in the eastern SPNA after 2005 reaches 0.75 and 0.9 °C/decade respectively. While the western SPNA continuously warms up since mid-1990s, except strong convection and cooling in 2008 and 2014^15^.

From 1995 to 1996, the mean temperature of the surface and subsurface eastern SPNA increase 0.75 and 0.6 °C respectively (Fig. 2c,d). This sudden warming is different from the western SPNA gradual warming. It has been attributed to preceding persisting strong positive NAO from 1989 to 1995 before sudden drop to negative NAO^23^. The mechanism involves both an enhanced northward transport of warm and salty water into the eastern SPNA by the North Atlantic Current (NAC) and the weakening SPG, which may be due to strengthened AMOC^19^, or the SPG weakening^16–18^. Prior “hiatus”, the relatively warmer and colder periods in both eastern and western SPNA are consistent with the NAO phase. The correlation coefficient between the eastern SPNA 200–700 m OHC and the NAO index is −0.54 (p = 0.0002), and for the western SPNA it is −0.61 (p = 0.00002). Numerical experiments verified the relation between the NAO related buoyancy forcing and the SPNA OHC^12^ as well. However, the correlation coefficients during the “hiatus” are low and insignificant, when the NAO changes signs more frequently. On multidecadal time scale, the AMO is in general consistent with the SPNA OHC at 0–200 m (Fig. 2e), with an earlier warmer period, followed by a cooler period and the recent warmer period.

## The Ocean Heat Content Decomposition

**Figure 3:**
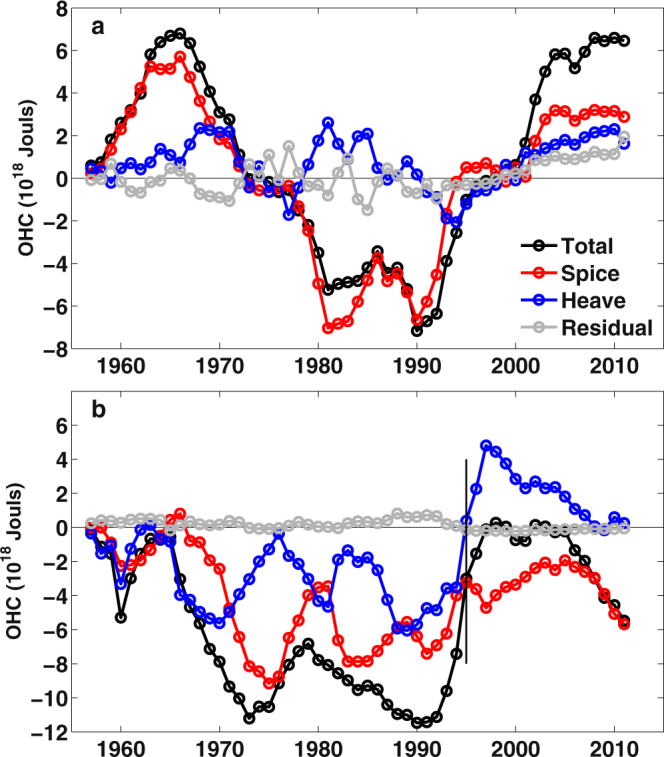
The along neutral surface decomposition of the ocean heat content changes referenced to the year 1956 in the SPNA. The evolution of the spice, heaving and residual components of the ocean heat content changes decomposition in the western SPNA (**a**) and eastern SPNA (**b**). The vertical thin black line points out the year 1995.

The temperature decomposition is applied in both the western SPNA and the eastern SPNA (Fig. 3). In the western SPNA, the temperature changes are mainly determined by the spice component through out most of the study time period (Fig. 3a), but during the “hiatus” period, the contribution by heaving component slightly increased. While in the eastern SPNA, the heaving and the spice components both show strong interannual variability (Fig. 3b), with one component contributes significantly more than the other from time to time, and the spice component mostly offsets the heaving component resulting in little OHC trend from 1998 to 2005. The findings here are seemingly contradicting the previous studies^21,22^, which showed a more dominating contribution by the heaving component in the North Atlantic or the SPNA. However, the decomposition in these studies is either a summation of the SPNA and the Subtropical North Atlantic, or the dominating heaving component in the SPNA resulted from a comparison between time periods 1957 to 1971 and 1997 to 2011^21,22^. The eastern SPNA has experienced a sudden warming in 1995^23^, and our results show that the heaving component is dominating during the sudden warming. The reversal of the warming trend in the eastern SPNA since 2005 has also been identified and attributed to the weakening of the AMOC and corresponding northward ocean heat transport^26^. In our study spice component indeed dominated the cooling since 2005.

We calculated correlation coefficient between the wind stress curl and the heaving components in western and eastern SPNA. The correlation between eastern SPNA heaving component and its mean wind stress curl is −0.5649 (p = 0.000929). This shows that the strengthening of the anticlockwise wind stress strengthens, then the subsurface isopycnal will sink and result in a negative temperature trend. The correlation between surface heat flux and the spice components are not good, because in the SPNA the ocean process is very important, and the lateral oceanic heat flux plays a more important role.

**Figure 4:**
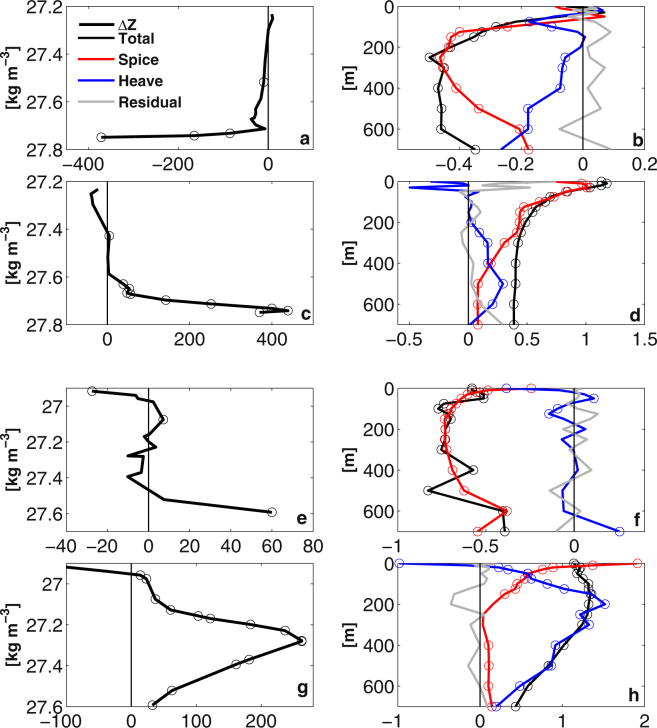
The vertical structure of the temperature decomposition decadal trends in the western and eastern SPNA. (**a**–**d**) are for the western SPNA, where (**a**) is the decadal trends of the vertical displacement (unit: meter) of the neutral surface during 1966–1975 cooling period, and (**b**) is the decadal trends (°C/decade) of the decomposed components during the same time period. (**c**) and (**d**) are the same as (**a**) and (**b**) but are the trends during 1991–2005 warming period. (**e**) and (**f**) are the same as (**a**) and (**b**) but for the eastern SPNA during 1966–1975 cooling. (**g**) and (**h**) are the same as (**e**) and (**f**) but are trends during the 1991–2000 warming period.

The vertical decomposition profile in the western SPNA also shows general domination of the spice component (Fig. 4b,d). The vertical displacement of the isopycnal in the deeper layer (Fig. 4a,c), and correspondingly the heaving component is enhanced below 500 m. The eastern SPNA temperature changes are dominated by spice component changes during the cooling (Fig. 4f), while during warming the heaving component is more important except at the surface 200 m where surface heat flux has more effect on the OHC (Fig. 4h). The vertical displacement is most significant around 400 m during the warming period (Fig. 4g). This is consistent with the vertically integrated decomposition in Fig. 3b, and generally both spice and heaving components can contribute significantly to the ocean heat content in the eastern SPNA.

## Mechanisms for Ocean Heat Content Trends

However, the forcings such as surface heat flux or remote heat flux due to ocean processes, or the wind stress forcing changes and subsequent gyre circulation changes cannot be readily attributed to the OHC. As elaborated, the pure warming, pure freshening and pure heaving scenarios are directly caused by certain forcings, but pure warming and pure freshening processes can induce heaving^22^. The buoyancy forcing induced heaving may offset or strengthen the wind stress induced heaving.

**Figure 5:**
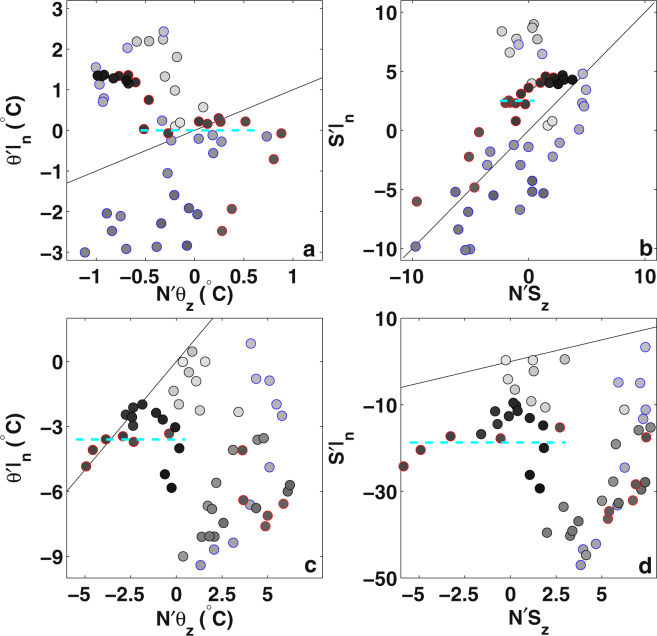
The hodograph of the spice and heaving components. (**a**) and (**b**) are the comparison of the spice and heaving components for temperature and salinity in the western SPNA, while (**c**) and (**d**) are the comparison for the eastern SPNA. Red circles indicate warming periods, and blue circles indicate cooling periods. The straight line has a slope of 1, circles along the straight line indicate changes due to pure buoyancy forcing. The dashed cyan line indicates when there is no salinity or temperature changes along the neutral surface. The color of the circles of increasing grey scales indicate evolution of yearly time.

Here, following previous methodology^20^, we use a hodograph to compare pairs of temperature or salinity changes along and across the neutral surface. When pure warming occurs, the salinity change along neutral surface should equal the salinity changes along the pressure surface. Similarly, when pure freshening occurs, the temperature changes along neutral surface should equal the changes along pressure surface. When pure heaving occurs there should be no temperature or salinity changes along the neutral surface. According to the hodograph analysis, the western SPNA ocean heat content change is mainly controlled by heat flux, since the correlation coefficient between the along and across neutral surface salinity change is about 0.6 (p = 0.000002) (Fig. 5b). Yet, in the eastern SPNA, there is no significant correlation between this pair of salinity components (Fig. 5c,d). Another notable event is that in the mid 1990s both eastern and western SPNA experienced stronger heaving, indicated by the small temperature and salinity changes along neutral surfaces.

In this study we have shown the strong decadal to multidecadal variability of the OHC in the SPNA. The 0–200 m OHC is more consistent with the AMO index. The NAO index has a high correlation with the 200–700 m OHC prior the recent “hiatus”, but cannot explain the OHC changes during the “hiatus”. Furthermore, during the “hiatus”, the temperature trends in the eastern and the western SPNA are opposite, indicating different physical mechanisms for the temperature changes. The decomposition of the temperature in the two regions of the SPNA shows a dominating spice component in the west while alternating domination of spice and heaving in the east. A closer inspection of the temperature trends of the vertical profiles during warming and cooling periods in the two regions further confirmed the spice domination in the west. Moreover for the eastern SPNA, the spice is more important during cooling, while heaving is more important during warming.

The warming is inhomogeneous in the SPNA during the “hiatus”, to be more specific the eastern SPNA is cooling down in the latter half of the “hiatus” after 2005, which has not been shown by previous “hiatus” study^3^. The western SPNA showed warming during the recent “hiatus” period, as well as the earlier one^3,24^. This consistency is due to the high correlation between the upper 700 m water temperature and the AMOC, and the slow-down of AMOC cause the deep Atlantic to warm up^2^. It is hypothesized that the SPNA salinity shifts on multidecadal time scales may have induced the fast deep penetration of heat in the Atlantic, resulting in the “hiatus”^3^. Therefore, the SPNA is still a key area to the “hiatus”, even though it takes up a much smaller area than the tropical Pacific Ocean, which bears more weight for subsurface heat uptake and surface cooling^7^.

The simple decomposition is actually powerful in identifying important physical processes during extreme events, such as the 1995 warming of the eastern SPNA, which has often been attributed to the gyre circulation changes^17^. Our results indeed show that the heaving component is dominating in 1995. The reversal of temperature trend since 2005 is directly due to a decreasing trend of the spice component, which is in line with the weakened AMOC and corresponding heat transport. Although it should be noted that further comparison of the salinity changes along and across neutral surface does not indicate pure warming, rather there could be a significant neutral surface undulation effect.

Finally it is important to note that the heaving and spice cannot be readily attributed to surface atmospheric buoyancy or wind forcing, and not even remote heat flux due to oceanic processes. As shown in this study, in order to attribute the temperature or ocean heat content change to certain forcing, the salinity and temperature changes along and across isopycnals should be compared. The ultra-high proportion of spice component does not necessarily mean heat flux induced pure warming or cooling process, unless the along and across isopycnal changes are approximately equivalent, such as the case in the western SPNA. Furthermore, the heaving component can be attibuted to wind stress only when the along isopycnal salinity and temperature changes are both relatively small. Even in this case, the wind stress curl has a complicated effect on the gyre isopycnal depths as shown in other studies^11,17^.

## Data and Methods

The global OHC from 1955 to 2013 is used to study the temperature variability and trend in the SPNA^26^. The mean temperatures of 0–200 m and 200–700 m are studied. The pentadal mean heat and salt content from World Ocean Atlas is used to decompose the OHC changes into two components^22^. One is referred to as the water mass change along the neutral surfaces, while the other is due to the undulation of the neutral surface and the vertical temperature gradient according to the 1st order tylor expansion^6,7,17,23^. The temperature changes along pressure level can be decomposed into the temperature change along the neutral surface and the temperature change due to the undulation of the neutral surface:

1 $${\frac{d\theta }{dt}|}_{z}={\frac{d\theta }{dt}|}_{{\rho }_{0}}+\frac{d\theta }{dz}{\frac{dz}{dt}|}_{{\rho }_{0}}$$

The December to March NAO index (https://climatedataguide.ucar.edu/climate-data/hurrell-north-atlantic-oscillation-nao-index-pc-based), an unsmoothed AMO index (https://climatedataguide.ucar.edu/climate-data/hurrell-north-atlantic-oscillation-nao-index-pc-based), and the Arctic sea ice extent index (ASIEI, http://nsidc.org/data/docs/noaa/g02135_seaice_index/#monthly_data_files) is used to investigate possible driving mechanisms for the SPNA OHC variability.

## Electronic supplementary material

Data Quality and Cross-Datasets Comparison
