# Supplementary material for: The Subpolar North Atlantic Ocean Heat Content Variability and its Decomposition
Source: Sci Rep. 2017 Oct 23;7:13748. doi: 10.1038/s41598-017-14158-6 (PMC5653740; doi:10.1038/s41598-017-14158-6)
Supplement: Supplementary file 1 — Data Quality and Cross-Datasets Comparison [file 41598_2017_14158_MOESM1_ESM.pdf]

The Subpolar North Atlantic Ocean Heat Content Variability and its Decomposition

Weiwei Zhang, Xiao-Hai Yan

Supplemental Materials

The analyzed EN4 4.2.0 data[1] is used to compare the temperature trends and decomposed components with the WOA data. The EN4 data used here have adopted the Levitus correction procedures[2]. The data is available at http://www.metoffice.gov.uk/hadobs/en4/data/en4-2-0/.

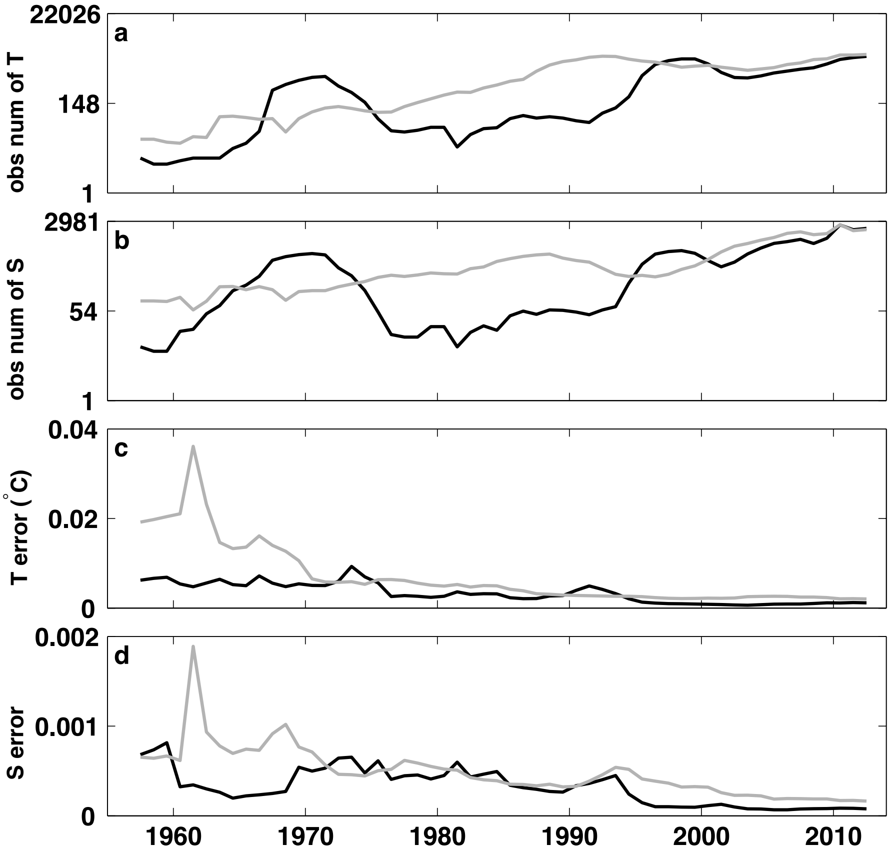

Figure S1 The total number of observations for potential temperature (a) and Salinity (b), and the mapping errors for potential temperature (c) and Salinity (d). The black curve is for western SPG, and the grey curve is for eastern SPG.

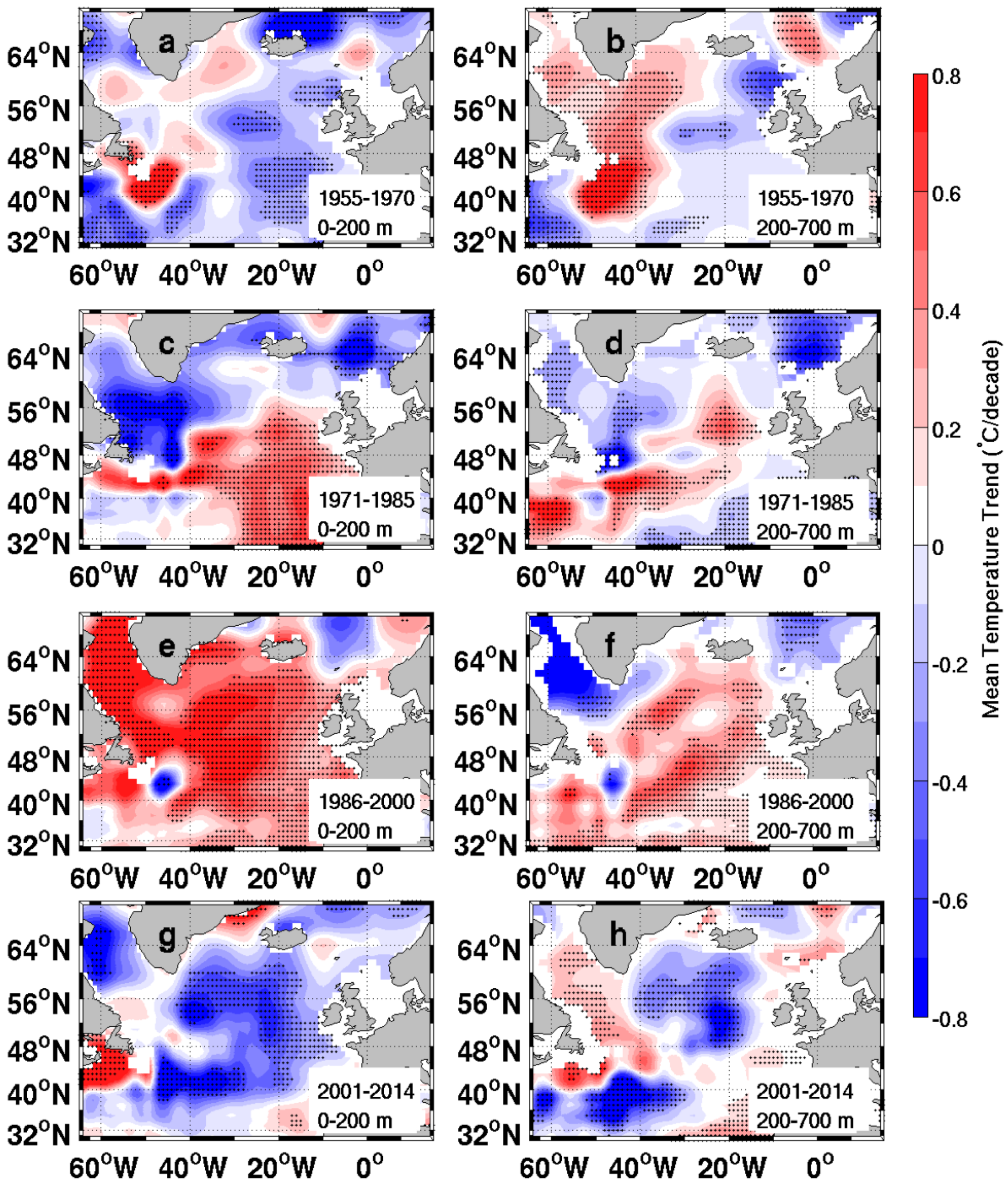

Figure S2 The vertically averaged EN4 potential temperature trend (unit: °C/decade) in the Subpolar North Atlantic. The linear trends of the temperature for different time periods are calculated for two depth ranges: 0-200 m and 200-700 m. The stipplings indicate that the fitted linear trend is above 95% confidence level. The map in this figure is created using the m_coast function from the m_map toolbox for Matlab (https://www.eoas.ubc.ca/~rich/map.html).

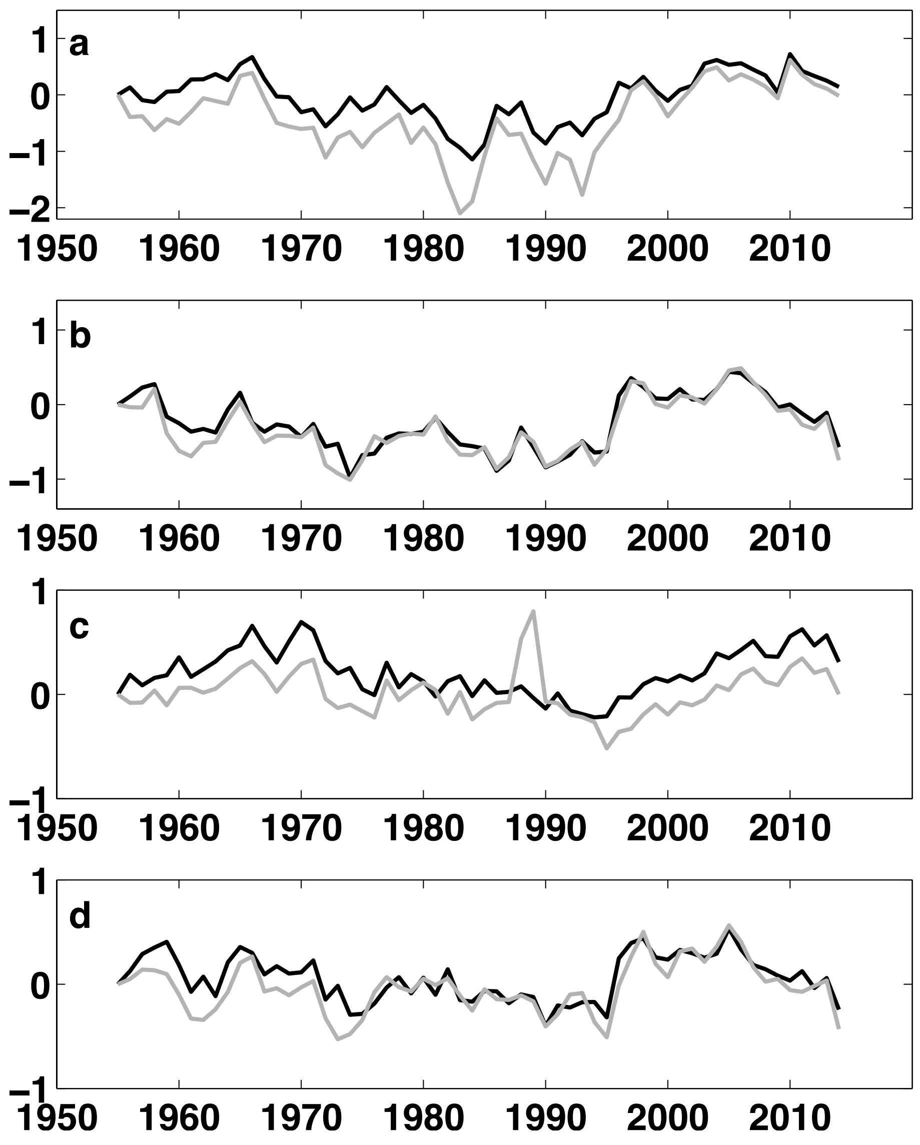

Figure S3 The mean temperature evolution of the western (a) and eastern (b) SPG at 0-200 m, and the temperature evolution of the western (c) and eastern (d) SPG at 200-700 m. Black curves are from WOA dataset, and the grey curves are from the EN4 dataset.

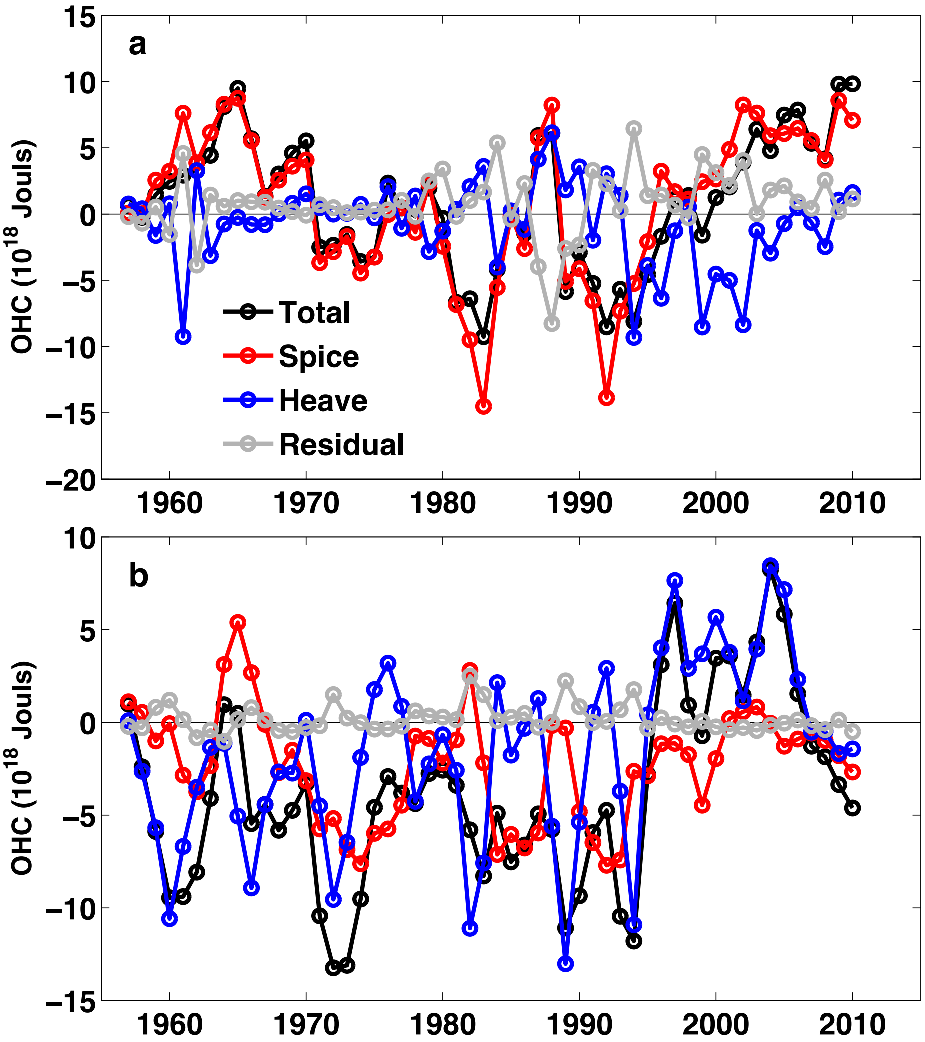

Figure S4 The along neutral surface decomposition of the ocean heat content changes referenced to the year 1956 in the SPNA. The evolution of the spice, heaving and residual components of the ocean heat content changes decomposition in the western SPNA (a) and eastern SPNA (b). The decomposed data here is the annual EN4 ocean subsurface temperature and salinity data.

References:

1. Good, S. A., M. J. Martin and N. A. Rayner, EN4: quality controlled ocean temperature and salinity profiles and monthly objective analyses with uncertainty estimates, *J. Geophys Res.: Oceans*, 118, 6704-6716 (2013)

2. Levitus, S. et al, Global ocean heat content 1955-2008 in light of recently revealed instrumentation problems, Geophys. Res. Lett., 36 L07608 (2009)
